# Evoking and Measuring Identification with Narrative Characters – A Linguistic Cues Framework

**DOI:** 10.3389/fpsyg.2017.01190

**Published:** 2017-07-13

**Authors:** Kobie van Krieken, Hans Hoeken, José Sanders

**Affiliations:** ^1^Centre for Language Studies, Radboud University Nijmegen, Netherlands; ^2^Utrecht Institute of Linguistics, Utrecht University Utrecht, Netherlands

**Keywords:** character, identification, linguistic viewpoint, narrative, perspective, reading experiences

## Abstract

Current research on identification with narrative characters poses two problems. First, although identification is seen as a dynamic process of which the intensity varies during reading, it is usually measured by means of post-reading questionnaires containing self-report items. Second, it is not clear which linguistic characteristics evoke identification. The present paper proposes that an interdisciplinary framework allows for more precise manipulations and measurements of identification, which will ultimately advance our understanding of the antecedents and nature of this process. The central hypothesis of our Linguistic Cues Framework is that identification with a narrative character is a multidimensional experience for which different dimensions are evoked by different linguistic cues. The first part of the paper presents a literature review on identification, resulting in a renewed conceptualization of identification which distinguishes six dimensions: a spatiotemporal, a perceptual, a cognitive, a moral, an emotional, and an embodied dimension. The second part argues that each of these dimensions is influenced by specific linguistic cues which represent various aspects of the narrative character’s perspective. The proposed relations between linguistic cues and identification dimensions are specified in six propositions. The third part discusses what psychological and neurocognitive methods enable the measurement of the various identification dimensions in order to test the propositions. By establishing explicit connections between the linguistic characteristics of narratives and readers’ physical, psychological, and neurocognitive responses to narratives, this paper develops a research agenda for future empirical research on identification with narrative characters.

## Introduction

Characters of fiction can evoke strong emotions in their readers. The Irish politician Daniel O’Connell is reported to have thrown Charles Dickens’ *The old curiosity shop* out of the window of a riding train after reading the part in which the heroine, little Nell, dies (Camden Hotten et al., 1870, p. 46). This kind of involvement with a character is usually referred to as “identification,” which crucially involves perspective-taking: perceiving the story events from the perspective of a character and while taking this character’s beliefs, values, and goals into account (e.g., Cohen, 2006).

There is abundant evidence, both anecdotal and scientific, that identification with a narrative character can have various and important effects on the reading experience. Additionally, identifying with a character can have important effects on the audience’s beliefs and attitudes in real life. Current research in this domain poses two problems. First: although researchers are aware that identification is a process and that the degree of identification and its immediate reactions in terms of perception, cognition, and affect, will vary during reading, the degree of identification and the accompanying emotions are mostly measured only *after* reading with questionnaires consisting of self-report items. The ability of these *ad hoc*, explicit measures to adequately capture complex narrative experiences such as immersion and identification has recently been subject of debate (Jacobs, 2015b; Dixon and Bortolussi, 2016; Kuiken, 2016). Second, it is not clear *how* the deployment of linguistic characteristics evokes identification. These two problems are interdependent. Measuring the degree of identification during reading requires knowledge of the perceptive, cognitive, and affective experiences that indicate the degree of identification, and this, in turn, requires knowledge on the linguistic structures that induce these dimensions of identification.

In recent years, models on reading experiences have been developed which integrate theories and methods from literary studies, linguistics and neuroscience to derive hypotheses which can be tested with offline as well as online measures, thus allowing for a more precise and comprehensive assessment of the impact of text-linguistic features on the processing of stories. For example, the *Neurocognitive Poetics Model of Literary Reading* (Jacobs, 2015c) posits that the background elements of a story (expressed in, e.g., familiar words) prompt a fast reading process that facilitates immersive experiences, whereas foregrounded elements (e.g., figurative language use) prompt a slow reading process that facilitates aesthetic experiences. The difference between both routes of processing is hypothesized to be discernible at the neuronal, affective-cognitive, and behavioral level. In support of the model, a study on poetry reception combining skin conductance measures with questionnaires showed that backgrounding elements affect readers’ emotional involvement whereas foregrounding elements such as style affect their aesthetic appreciation of poems (Lüdtke et al., 2014).

The present paper aims to build on this ‘new’ scientific study of literature by presenting a multidimensional framework that focuses on identification experiences taking place while reading narratives. The framework integrates linguistic research on the representation of perspective in (narrative) discourse and psychological research on identification. Our central hypothesis is that identification with a narrative character is a multidimensional experience for which different dimensions are evoked by different linguistic cues that give expression to various aspects of the narrative character’s viewpoint. The framework comprises several sub-hypotheses. We present an agenda for future research testing these hypotheses with behavioral and neuroscientific measures, with the ultimate aim to advance our understanding of identification by employing more precise manipulations and measurements of this process.

We will start with a literature review of identification, leading to an empirically testable conceptualization of identification, which will be followed by the introduction of our multidimensional Linguistic Cues Framework that includes an inventory of the various linguistic structures writers can use to evoke identification with narrative characters. We conclude by presenting an agenda for future research assessing the impact of these techniques on identification.

## Literature Review

Perhaps the most characteristic aspect of a (good) story is its capacity to engage its audience, sometimes to the extent that the story world becomes more real to them than the actual world in which they are reading or watching the story. This experience has been called “transportation” (Gerrig, 1993; Green and Brock, 2000, 2002), “absorption” (Slater and Rouner, 2002), “narrative engagement” (Busselle and Bilandzic, 2009), or “immersion” (Jacobs, 2015a). So far, immersion appears the only process that has been examined with neurocognitive methods. For example, in an fMRI study on immersive experiences taking place while reading emotion-laden versus neutral passages of Harry Potter, the emotion-laden passages resulted in stronger correlations between blood oxygenation level dependent (BOLD) signals in neural substrates associated with empathy on the one hand and *post hoc* immersion ratings on the other (Hsu et al., 2014).

Yet, most research on readers’ engagement with stories has been conducted in the humanities and social sciences. Studying engagement with audiovisual narratives, Busselle and Bilandzic (2009) showed that this experience is multidimensional, consisting of focusing one’s attention on the events in the story world, the feeling of being present at these events, and the experiencing of emotions as a result of the story characters’ vicissitudes. The experience could be disrupted when the audience had problems in following the thread of the story. Similar dimensional patterns have been found studies employing written narratives (e.g., De Graaf et al., 2009, 2012; Hoeken and Fikkers, 2014).

One important dimension of engagement is the experience of feeling present at the events, or as Oatley (1999, p. 445) puts it: the audience becoming “an unobserved observer in scenes of the lives of characters in the story world. He or she stands in their bedrooms, hovers at their dining tables, drives with them in their cars.” This type of experience is similar to what Busselle and Bilandzic (2009) call “narrative presence” and what Jacobs (2015a) refers to as “spatial immersion.” Oatley (1999, p. 445) contrasts this type of involvement with identification, which he defines as “the reader takes on the protagonist’s goals and plans.” As a result of the audience considering the character’s goals as important themselves, it will experience emotions “as these plans meet vicissitudes” (Oatley, 1995, p. 66). The valence of these emotions depends on whether the character succeeds or fails to attain his or her goals, with success leading to experiencing joy and contentment and failure to sadness or anger. The intensity of these emotions depends on the extent to which the readers identified with the character; the more they care about a character, the more they will rejoice at the character’s successes or mourn its failures. From this perspective, the intensity of Daniel O’Connell’s response to the death of little Nell reflects the deep interest he held in her welfare.

Although related, narrative presence and identification are distinguishable aspects of narrative engagement. Tal-Or and Cohen (2010) manipulated identification with a character independently of manipulating the feeling of being present. Level of identification proved susceptible to the character being portrayed as sympathetic or unsympathetic; the level of transportation was manipulated by providing information on a future event (thereby increasing suspense) or on an event in the past. Both manipulations proved successful in manipulating the level of identification and narrative presence independently of each other. Sestir and Green (2010) achieved differential levels of identification by instructing participants to either identify with the main character or to observe the events as an independent observer; they manipulated the level of narrative presence by instructing (other) participants to either focus on the events as if experiencing the events themselves or to focus on the color scheme used.

Identifying with a character can have important effects on the audience’s real life beliefs and attitudes. For instance, identifying with characters from a television series influences risk perceptions of teen pregnancy and subsequent intentions to have safe sex (Moyer-Gusé and Nabi, 2010) as well as the intention to talk about sexually transmitted infections with friends (Moyer-Gusé et al., 2011). Similarly, identifying with characters in movies has been shown to mediate the impact of these movies on the audience’s beliefs (Igartua, 2010; Igartua and Barrios, 2012), social distancing toward people with a mental illness (Caputo and Rouner, 2011), and attitudes toward the death penalty (Till and Vitouch, 2012). Finally, identifying with characters in written stories has had participants adapt their opinions to those of the character (e.g., De Graaf et al., 2012), even if the character’s opinion goes against the readers’ interest (Hoeken and Fikkers, 2014), and can even moderate attitudes toward highly controversial issues such as the Israeli-Palestinian conflict (Cohen et al., 2015). This raises the question as to what factors determine with which character and to what extent the audience identifies itself?

### Determinants of Identification

Cohen (2006) distinguishes between two classes of determinants of character identification: character based factors and storytelling techniques. Character based factors concern the extent to which the audience perceives a character as similar to itself and the extent to which it considers the character likeable or sympathetic. Several scholars have suggested that identification with a character is evoked by the extent to which an audience member considers him or herself as similar to the character (Slater and Rouner, 2002; Cohen, 2006; Brown, 2015).

#### Character-Based Factors

In several studies, positive correlations between perceived similarity and identification were obtained (e.g., Moyer-Gusé and Nabi, 2010; Pinkleton et al., 2010; Murphy et al., 2013). In De Graaf (2014), similarity was manipulated by having students read a story about a student who lived with her parents in one version of the story, or in student housing in a second version. Participants indicated whether they themselves lived with their parents or in student housing. For matching living conditions, participants perceived themselves as more similar to the protagonist than for mismatched conditions. However, matching or mismatching living conditions did not influence the level of identification.

Likewise, Andsager et al. (2006) developed two stories that only differed with respect to the protagonist drinking alcohol or not. They also assessed whether participants themselves drank alcohol. If the (non)drinking behavior of the participant matched that of the protagonist, they perceived themselves as more similar to the protagonist. On a similar note, McKeever (2015) found that participants perceived themselves as more similar to a person in a news story if that person was a student at the same university than if he was not. Neither Andsager et al. (2006) nor McKeever (2015) measured identification but they both found perceived similarity to predict the message’s persuasiveness.

Finally, Hoeken et al. (2016) had participants read a story in which one of the characters was a lawyer. Half of their participants were Law school students, the other half were Humanities students. Not only did the Law students consider themselves more similar to the lawyer than the Humanities students did, they also identified more strongly with this character. In a second study, a similar effect was obtained when Med school students and Humanities students read a story in which one of the characters was a General Physician. The fact that De Graaf (2014) did not find effects of similarity in living conditions on identification may be caused by how similarity was evoked. Cohen (2006, p. 188) claims that psychological similarity (e.g., having similar attitudes or personality traits) is more important for identification than demographic similarity (such as gender and age). De Graaf’s (2014) “living conditions” manipulation can be considered a demographic similarity, whereas the “alcohol drinking” or “having a similar education” manipulations may be more related to psychological similarities.

The second character-based driver of identification is considered a character’s likeability (Cohen, 2006): the audience is more inclined to identify with a character they consider to be “good,” which implies that the character holds, and acts upon the same norms and values upheld by the audience member. Raney (2004) suggests that audiences are tirelessly evaluating the morality of a character’s ideas and actions. When they approve of these ideas and actions, they are more likely to consider the character’s goals as desirable themselves and the actions needed for attaining these goals as justified. Manipulating a character’s likeability by portraying it in a more or less favorable way has indeed led to changes in the level of identification with the characters (e.g., Tal-Or and Cohen, 2010; Hoeken and Sinkeldam, 2014).

Especially when identification is driven by perceived ‘psychological’ similarity as is argued by Cohen (2006), similarity and likeability are related factors. Correspondence between the character’s values and personality and the audience’s values and personality is likely to result in the character’s actions being in line with the audience’s normative preferences. One could argue that likeability is evoked by the evaluation of a character’s actual actions and ideas as being virtuous or good, whereas similarity holds the promise that the character’s actions and idea probably will be in accordance with those of the audience members.

#### Viewpoint Techniques

Apart from similarity and likability as character-based drivers of identification, Cohen (2006) refers to storytelling techniques as a means to evoke identification. A highly influential technique in this respect is the strategic employment of “point of view” or “perspective”: from whose perspective do we experience the events related in the story? In other words: does the audience have privileged access to the perceptions, evaluations, and goals of a character? This kind of access is important because it enables the audience to take this character’s perspective. Perspective-taking is considered a core aspect of identification. Brown (2015, p. 275–276), for instance, states that “conforming to the identity of a persona includes taking on his or her perspective”; Cohen (2006, p. 184) defines identification as “an imaginative process through which an audience member assumes the identity, goals, and perspective of a character.” Oatley (1999) argues that point of view can turn the audience from an invisible witness to the events into an audience that considers the goals and stakes of a specific character as more important when the perspective of that character to the story events is taken.

Leech and Short (2007, p. 221) argue that the “very exposure […] to a character’s point of view – his thoughts, emotions, experience – tends to establish an identification with that character, and an alignment with his value picture.” Several studies attest to the importance of point of view for identification. De Graaf et al. (2012) manipulated the extent to which participants identified with characters having conflicting opinions by favoring the perspective of either one character or the other. Participants typically identified more strongly with the character whose thoughts and emotions were represented regardless of the opinion held by that character. Hoeken and Fikkers (2014) found a similar effect of representing a character’s point of view even if the character held an opinion that went against the readers’ best interests; students identified more strongly with a character claiming that a tuition raise was desirable than with a character opposing a tuition raise if the events were related from the point of view of the former character.

Hoeken et al. (2016) pitted the impact of a character based driver of identification, similarity, against that of perspective. Whereas Law school students identified more strongly with a lawyer defending a murderer than with the victim’s widow, this effect was reversed when the story was told from the perspective of the widow. They found a similar effect for a story about a conflict between a General Physician and the son of an elderly patient: Med school students identified more strongly with the GP than with the son unless the story was told from the perspective of the son. These findings show that the representation of a character’s point of view can have readers identify with a certain character even in the presence of a more similar character.

In narrative discourse, a fundamental choice in perspective is the grammatical person of the protagonist, first or third person: an “I” as narrative character, or a third person character “he” or “she.” Connected is the choice between narrative positions construing a narrator who either plays a role in the narrative events (intradiegetic), or merely functions as a distant narrator (extradiegetic; Genette, 1980). By definition, an “I”-character is an intradiegetic narrator, relating his or her own experiences and those of other characters, whereas a third person narrative has an extradiegetic narrator by definition. First person narratives invite readers to represent the perspective from their personal, spatial body perspective (Brunyé et al., 2009). In text processing, the grammatical first person guides the reader toward identification with the “I”-character, who is the deictic center in which the text is referentially grounded; by default, speakers implicitly perceive and think from this center (Graesser et al., 2002). Readers appear to use “self” as an anchor in organizing information (D’Ailly et al., 1995); the “I”-perspective seems evolutionary primal, and neuropsychological research of visual perspectives shows that first person perspective is processed in a neurological different way than third person perspective (Vogeley et al., 2004).

The choice between first and third person narrative perspective is thus fundamental in each narrative and in the majority of the studies discussed above, this perspective was manipulated by varying first person versus third person characters. Although readers were found to identify more strongly with first person than third person characters, this does by no means imply that they cannot, or only to a low degree, identify with third person characters. On a conceptual level, first and third person characters do not essentially differ in degree of subjectivity: both can function as explicit subjects of consciousness in the narrative discourse and can be applied equally well to depict perceptions, emotions, and evaluations from an internal perspective (Sanders et al., 2012). Thus, similar to first person narrations, third person narrations allow for great variability in terms of viewpoint representation, ranging from relatively subtle representations of a character’s point of view to full-blown representations of a character’s perceptions, beliefs, and desires.

While little is known about the impact of these various linguistic viewpoint indicators, a recent study showed how even subtle viewpoint phenomena in third person stories can influence readers’ identification (Van Krieken and Sanders, 2017); more specifically, the use of pronouns rather than nouns to refer to a character was found to increase readers’ emotional and cognitive identification with that character, but not their spatiotemporal identification. This finding further supports the theoretically established link between linguistic viewpoint and identification (e.g., Leech and Short, 2007; Farner, 2014); moreover, it suggests that different dimensions of identification might be influenced by different types of linguistic viewpoint cues. In order to arrive at a more thorough understanding of both the antecedents and the nature of identification with narrative characters, studies are called for to assess the connection between various distinctive types of viewpoint cues, in both first person and third person stories, and various distinctive dimensions of identification. We propose that research in this direction can benefit from an interdisciplinary approach, by integrating linguistic and literary accounts of viewpoint construction in narrative discourse and psychological and neurocognitive methods to measure identification.

## Multidimensional Linguistic Cues Framework

Our approach is in line with recently developed models and theories on the study of literary experiences. Burke (2015), for example, argues that stylistic analyses of stories enable the formulation of hypotheses about the processing of stories which can be empirically tested with neurocognitive methods. Similarly, Willems and Jacobs (2016) argue that studying processes of immersion and aesthetic appreciation requires a thorough understanding of the linguistic finesses of stories. These finesses include metric, phonological, morpho-syntactic, and semantic features at the sublexical, lexical, interlexical, and supralexical levels of discourse (Jacobs, 2015a; Jacobs et al., 2016b).

Whereas these previously developed models aim to account for a variety of reading experiences, the present paper presents a multidimensional framework that focuses exclusively on the experience of identification with narrative characters. In line with previous research (e.g., Cohen, 2001, 2006; Igartua and Barrios, 2012), we conceptualize identification as a multidimensional experience of mental enactment that evolves from processes in which the reader takes over the central narrative subject’s perspective. Perspective is here considered to cover aspects related to this subject’s experience of narrative events and situations from a particular point in time and space as well as aspects related to the subject’s world view. All of these aspects are crucial in the reader’s construction of a situation model, i.e., the mental representation of the story which includes a representation of the spatial lay-out, temporal order, people, objects, and goals (Zwaan et al., 1995; Zwaan and Radvansky, 1998; Zwaan, 1999). Developing and updating a situation model is guided by linguistic elements that cue the construal of various dimensions of the model, facilitating the reader’s transformation into an “immersed experiencer” (Zwaan, 2004).

**FIGURE 1:**
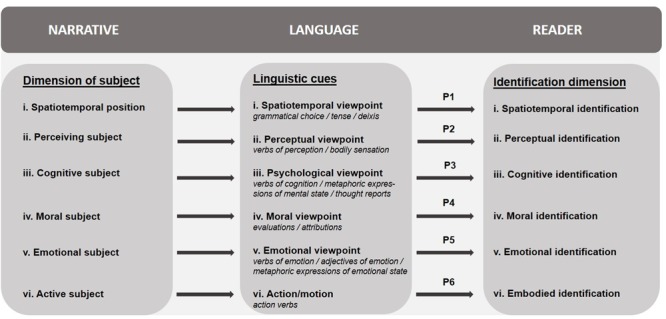
Multidimensional Linguistic Cues Framework of identification.

Accordingly, we distinguish various dimensions of the subject that can be expressed in narrative discourse, either in isolation or in combination with each other, each corresponding to a specific dimension of identification. **Figure 1** below presents a multidimensional Linguistic Cues Framework that connects the various dimensions of the subject to the various dimensions of identification by means of linguistic cues that give expression to the narrative character’s viewpoint and actions. The proposed causal connection between linguistic aspects of the narrative and identification processes taking place in readers constitutes the central hypothesis of the framework. This hypothesis is based on the assumption that in order to take over the perspective of a character and identify with a character, readers must be presented with that character’s perspective. Given the multidimensional nature of identification, as well as the wide range of linguistic cues expressing various aspects of the character’s viewpoint (e.g., Van Krieken et al., 2016), the model comprises six sub-hypotheses. These hypotheses are represented in the framework as propositions P1–P6 and will be elaborated upon in the following sections.

### Linguistic Cues Guiding the Six Identification Dimensions

In this section we will elaborate on the various identification dimensions and discuss how each dimension of the narrative subject is construed by the use of specific linguistic cues expressing the subject’s viewpoint and actions. **Table 1** provides an overview with examples of these cues in both first person and third person stories.

**Table 1:** Various dimensions of the narrative subject and their linguistic characteristics.

Dimension of subject	Linguistic characteristics	Operationalization with third person character (extradiegetic narrator)	Operationalization with first person intradiegetic narrator (=character)
(i) Spatiotemporal position	(1a) Grammatical choices (1b) Tense (1c) Deictic elements	(1a) He took her arm and walked her to the garden. (1a’) She was taken by the arm by him and was walked to the garden. (1b) She stepped outside. She walked into the garden. (1b’) She stepped outside. She had walked in the garden before. (1b”) She steps outside. She walks into the garden. (1c) She stepped outside. She had walked here/in this garden before. (1c’) She stepped outside. She had walked there/in that garden before.	(1a) He took my arm and walked me to the garden. (1a’) I was taken by the arm by him and was walked to the garden. (1b) I stepped outside. I walked into the garden. (1b’) I stepped outside. I had walked in the garden before. (1b”) I step outside. I walk into the garden. (1c) I stepped outside. I had walked here/in this garden before. (1c’) I stepped outside. I had walked there/in that garden before.
(ii) Perceiving subject	(2a) Verbs of bodily sensation (2b/c) verbs of perception	(2a) She stepped outside. The sunlight hurt her eyes. (2b) She felt wobbly. Slowly she stepped outside. (2c) She looked outside. The garden was filled with purple flowers. (2c’) She stepped outside. The garden was filled with purple flowers.	(2a) I stepped outside. The sunlight hurt my eyes. (2b) I felt wobbly. Slowly I stepped outside. (2c) I looked outside. The garden was filled with purple flowers. (2c’) I stepped outside. The garden was filled with purple flowers.
(iii) Cognitive subject	(3a) Verbs of cognition (3b) Metaphoric expressions of mental state (3c) Thought reports	(3a) She thought of the garden. It would be filled with purple flowers by now. (3b) She set her mind to the garden. It needed plowing and sowing. (3c) She stepped outside. “Did I see this garden before?” she wondered. (3c’) She stepped outside. She wondered if she had seen this garden before. (3c”) She stepped outside. Hadn’t she seen this garden before?	(3a) I thought of the garden. It would be filled with purple flowers by now. (3b) I set my mind to the garden. It needed plowing and sowing. (3c) I stepped outside. “Did I see this garden before?” I wondered. (3c’) I stepped outside. I wondered if I had seen this garden before. (3c”) I stepped outside. Hadn’t I seen this garden before?
(iv) Moral subject	(4a) Evaluations (4b) Attributions (& beliefs, desires)	(4a) The garden was filled with purple flowers. Awfully old-fashioned. (4a’) She approved of choosing orange flowers, but not purple ones – they were all genetically manipulated. (4b) The garden had once been filled with delightful purple flowers, but one day the terrible youngsters broke into her garden. All flowers were trampled. The surroundings had been left barren, as they were to this day. It was all the fault of these sloppy parents who failed to give their children a strict upbringing. If only she had the energy to start all over again.	(4a) The garden was filled with purple flowers. Awfully old-fashioned. (4a’) I approved of choosing orange flowers, but not purple ones – they were all genetically manipulated. (4b) The garden had once been filled with delightful purple flowers, but one day the terrible youngsters broke into my garden. All flowers were trampled. The surroundings had been left barren, as they were to this day. It was all the fault of these sloppy parents who failed to give their children a strict upbringing. If only I had the energy to start all over again.
(v) Emotional subject	(5a) Verbs of emotion (5b) Adjectives of emotion (5c) Metaphoric expressions of emotional state	(5a) She loved the garden. It was filled with purple flowers. (5a’) She hated the garden. It was filled with purple flowers. (5b) She was enlightened by the garden. It was filled with purple flowers. (5b’) She was abhorred by the garden. It was filled with purple flowers. (5b”) She was scared to enter the garden. It was too dark. (5c) She was moved by the colors of the garden. (5c’) The colors of the garden hurt her eyes.	(5a) I loved the garden. It was filled with purple flowers. (5a’) I hated the garden. It was filled with purple flowers. (5b) I was enlightened by the garden. It was filled with purple flowers. (5b’) I was abhorred by the garden. It was filled with purple flowers. (5b”) I was scared to enter the garden. It was too dark. (5c) I was moved by the colors of the garden. (5c’) The colors of the garden hurt my eyes.
(vi) Active subject	(6) Action verbs (& events)	(6) She worked in the garden day and night. With great effort, she plowed the ground and sowed the flowers. Yet in the Summer, vagabonds came to the area and stole all flowers. They left her with an emptied garden. The next Spring, she started all over again.	(6) I worked in the garden day and night. With great effort, I plowed the ground and sowed the flowers. Yet in the Summer, vagabonds came to the area and stole all flowers. They left me with an emptied garden. The next Spring, I started all over again.

#### Spatiotemporal Identification

The most basic dimension of identification is spatiotemporal identification, a process through which readers adopt the character’s physical location in time and space as a vantage point from which to interpret temporal and spatial information. In any story, characters move within a demarcated space within a demarcated period of time. Descriptions of a character’s movements and actions are necessarily tied to the character’s spatiotemporal viewpoint. In this line of reasoning, any narrative description referring to a character can be thought of as evoking a certain degree of spatiotemporal identification. A study by Morrow et al. (1987) lends support to this assumption. This study showed that after reading a narrative, people were faster to respond to the names of objects depending on how closely located these objects were to the character in the narrative world. These results indicate that in the construction of a mental representation of a story, readers align their viewpoints with the viewpoints of characters that play a part in it. Spatiotemporal identification is therefore considered a default mode of identification.

Spatiotemporal identification might be further enhanced by linguistic signals of a character’s spatiotemporal viewpoint. Linguistic elements that are likely to affect this dimension of identification include grammatical choices, verb tense, and deictic elements. For example, the metaphoric camera is typically located with characters in grammatical subject (rather than grammatical object) position of a clause (Van Hoek, 2007; Van Krieken et al., 2015). In examples (1a) and (1a’), the propositional content is identical, but in (1a) the scene is viewed from the male character’s point of view whereas in (1b) the scene is viewed from the female character’s point of view:

(1a) He took her arm and walked her to the garden.
(1a’) She was taken by the arm by him and was walked to the garden.

Choice of grammatical subject can thus be thought of as affecting readers’ spatiotemporal identification in the sense that they view the described scene through the eyes of the character in subject position (Van Hoek, 2007, p. 900).

Spatiotemporal identification might also be affected by tense choices. Compared to the past tense (1b), the plusquamperfectum (1b’) decreases the temporal distance between character and reader, while the present tense (1b”) virtually collapses the tenses of character and reader (Dancygier, 2012; Van Krieken et al., 2016); both tenses evoke a stronger suggestion of internal perspective than the past tense.

(1b) She stepped outside. She walked into the garden.
(1b’) She stepped outside. She had walked in the garden before.
(1b’’) She steps outside. She walks into the garden.

Finally, spatiotemporal identification can be expected to be influenced by deictic elements, i.e., elements that are meaningful only from a certain point in time and space. Specifically, the use of proximal deictics (*here, this, now*) is likely to result in stronger identification than the use of distal deictics (*there, that, then*), since the former such as in (1c) create a sense of nearness whereas the latter such as in (1c’) create a sense of distance.

(1c) She stepped outside. She had walked here/in this garden before.
(1c’) She stepped outside. She had walked there/in that garden before.

Thus, our first proposition is formulated as follows:

P1: Linguistic cues of a narrative character’s spatiotemporal viewpoint enhance the reader’s spatiotemporal identification with that character.

#### Perceptual Identification

Perceptual identification constitutes the second dimension of identification. This dimension refers to the process in which readers adopt the character’s perceptual perspective and mentally represent what the character sees, hears, and physically experiences. This dimension of identification can be affected by linguistic references to a character’s perceptions and sensations.

In many stories, large parts of the events are rendered through the visual, auditory, and tactile perceptions of characters. Readers represent a character’s perceptual perspective by drawing inferences about the relation between the perceiver and that what is being perceived (Bortolussi and Dixon, 2003). This relation can be linguistically expressed in an explicit way, for example by the use of verbs of bodily sensation (2a, 2b) or verbs of perception (2c):

(2a) She stepped outside. The sunlight hurt her eyes.
(2b) She felt wobbly. Slowly she stepped outside.
(2c) She looked outside. The garden was filled with purple flowers.

Note that even when a verb of perception is absent such as in (2c’) below, the second sentence is an observable state rather than an action or event and might therefore be interpreted as a perception of the character as well (Brinton, 1980; Caenepeel, 1989; Farner, 2014):

(2c’) She stepped outside. The garden was filled with purple flowers.

Instances like these have been termed *free indirect perceptions* (Palmer, 2004), i.e., perceptions which could be attributed to either the narrator or the character. This ambiguity allows for a certain degree of perceptual identification with the character. Nevertheless, we expect identification to be strongest in instances such as (2a-c) because of their explicit reference to the character’s sensations and perceptions (c.f. Sanders, 1994). This leads to our second proposition:

P2: Linguistic cues of a narrative character’s perceptual viewpoint enhance the reader’s perceptual identification with that character.

#### Cognitive Identification

Through cognitive identification, readers come to share a character’s mindset, i.e., the character’s thoughts, expectations, aims, intentions, etcetera. Cognitive identification shares some similarity with cognitive empathy, a concept which refers to a person’s ability to draw inferences about another person’s thoughts and intentions (Brink et al., 2011). An important theoretical difference is that cognitive empathy is characterized by the *recognition* of another person’s mindset, whereas cognitive identification refers to the *adoption* of another person’s mindset. The self-other distinction is, thus, more pronounced in empathic responses compared to identification. When applied to narrative discourse, both processes involve the mental representation of the character’s mental state, but only cognitive identification involves the reader also taking over the character’s expectations, aims, and intentions.

Cognitive identification is likely to be influenced by linguistic elements that signal the character’s psychological viewpoint, which can be linguistically encoded by so-called verbs of cognition, for example:

(3a) She thought of the garden. It would be filled with purple flowers by now.

Metaphoric expressions of a character’s mental state constitute a second group of psychological viewpoint signals, for example:

(3b) She set her mind to the garden. It needed plowing and sowing.

Finally, a character’s psychological viewpoint can be represented by reporting, through various means of verbalization, his or her thoughts. Canonical modes of thought representation are direct thoughts, indirect thoughts, and free indirect thoughts (e.g., Semino and Short, 2004):

(3c) She stepped outside. “Did I see this garden before?” she wondered.
(3c’) She stepped outside. She wondered if she had seen this garden before.
(3c’’) She stepped outside. Hadn’t she seen this garden before?

Direct thoughts such as (3c) represent a character’s psychological viewpoint most explicitly since they verbalize thoughts verbatim. Indirect (3c’) thoughts, by contrast, do not depict thoughts in a literal way but paraphrase to larger or lesser extent the inner voice of the character (Van Krieken and Sanders, forthcoming), while thoughts represented in the free indirect mode lead to the inner voice of the character becoming intertwined with the narrator’s voice (Sanders and Redeker, 1996; Semino and Short, 2004).

Based on the literature discussed above, our third proposition is formulated as follows:

P3: Linguistic cues of a narrative character’s psychological viewpoint enhance the reader’s cognitive identification with that character.

#### Moral Identification

Moral identification can be conceptualized as a process through which readers adopt the beliefs, goals, moral values, and evaluative attitudes of a character. Among the linguistic elements that signal a character’s morality are evaluations, attributions, memories, and desires:

(4a) The garden was filled with purple flowers. Awfully old-fashioned. She liked the orange flowers, but not the purple ones that were genetically manipulated.
(4b) The garden had once been filled with delightful purple flowers, but 1 day the terrible vagabonds had got to the area and all flowers were stolen, leaving the surroundings barren as they were to this day. If only she were strong enough to start all over again.

The evaluation of narrative events and situations by the moral subject in terms of norms, values, and goals may give rise to emotions in readers, as the cognitive appraisal of objects, acts and persons is theorized to evoke such emotions (Smith and Ellsworth, 1985). Hence our fourth proposition:

P4: Linguistic cues of a narrative character’s moral viewpoint enhance the reader’s moral identification with that character.

#### Emotional Identification

Emotional identification occurs when readers adopt a character’s feelings and emotions. This dimension bears some resemblance to the concept of affective empathy, which refers to a person’s ability to identify another person’s emotions and respond to those emotions (Brink et al., 2011). However, similar to the distinction between cognitive identification and cognitive empathy, there is an important theoretical difference between emotional identification and affective empathy. Whereas affective empathy denotes the *recognition* of other persons’ feelings, emotional identification is characterized by the *adoption* of the feelings of another. For example, when a character is sad, the reader might feel sympathetic (signaling affective empathy) or sad (signaling emotional identification).

Emotional identification could be guided by linguistic representations of a character’s emotional viewpoint. Verbs of emotion, for example, provide narrators with various possibilities to express characters’ positive (5a) or negative (5a’, 5a”) feelings:

(5a) She loved the garden. It was filled with purple flowers.
(5a’) She hated the garden. It was filled with purple flowers.

Similarly, a character’s emotional viewpoint can be expressed by means of adjectives:

(5b) She was enlightened by the garden. It was filled with purple flowers.
(5b’) She was abhorred by the garden. It was filled with purple flowers.
(5b’’) She was scared to enter the garden. It was too dark.

Metaphoric expressions of a character’s emotional state constitute a third category of emotional viewpoint representation, for example:

(5c) She was moved by the colors of the garden.
(5c’) The colors of the garden hurt her eyes.

Our fifth proposition is formulated as follows:

p5: Linguistic cues of a narrative character’s emotional viewpoint enhance the reader’s emotional identification with that character.

Specific expressions of the narrative subject’s spatiotemporal, perceptual, cognitive, moral, and/or emotional perspective, collaboratively construe the inner world of this protagonist; together, they sketch this character’s *landscape of consciousness* (Bruner, 1986). Some narratives favor the representation of this inner landscape over the narrative event line, whereas other narratives will foremost sketch the *landscape of action* (Bruner, 1986), in which the narrative subject acts and takes part in events.

Importantly, the narrative action is *not less* subjective than the inner consciousness, but a *different way* of conceptualizing the subject: the narrative action enables readers to represent the acts and events from a subjective viewpoint. As is the case in landscape of consciousness, the subjective viewpoint of the landscape of action is rooted in the spatiotemporal perspective; reference and grammar together point out which subject(s) are central. The narrative subject(s) partaking in the action are represented by readers in the sixth and final dimension of identification: embodied identification.

#### Embodied Identification

Through embodied identification, readers mentally simulate performing the character’s actions and motions, co-experiencing the events the character goes through. Both behavioral and neuroimaging studies have shown that language comprehension involves and most likely even requires activation of motor systems, meaning that actions described in language are mentally simulated without being physically executed (for an overview, see Fischer and Zwaan, 2008). When processing narrative discourse, action simulation is prompted by linguistic representations of a character’s actions and motions (Speer et al., 2009), e.g.:

(6) She worked in the garden day and night. With great effort, she plowed the ground and sowed the flowers. Yet in the Summer, vagabonds came to the area and stole all flowers. They left her with an emptied garden. The next Spring, she started all over again.

Our final proposition is formulated as follows:

P6: Linguistic cues of a narrative character’s action and motions enhance the reader’s embodied identification with that character.

### Measuring Effects of Linguistic Viewpoint Cues on Identification

The above inventory shows that narrators can employ a range of highly diverse linguistic resources, either stressing the subjective consciousness or the subjective line of narrative events, to describe narrative events from the viewpoint of first person and third person characters. To date, only few studies have examined the effects of viewpoint cues on readers’ responses. Van Peer and Pander Maat (2001), for instance, manipulated perspective by including or excluding a character’s thoughts into a story. Similarly, Hakemulder and Koopman (2010) made a comparison between stories with and stories without speech and thought reports in the Free Indirect Style. Sanders and Redeker (1993) manipulated perspective in a more subtle way, by comparing stories with versus without verbs of sensory perception (*see*) and verbs referring to cognitive states (*realize*).

Notably, however, none of these previous studies measured readers’ identification with narrative characters. In this section, we discuss possibilities to assess the relation between the various types of linguistic viewpoint cues and the various identification dimensions by employing (neuro)psychological methods. Such methods can complement commonly used methods which assess identification by means of self-report items (e.g., Igartua, 2010; Tal-Or and Cohen, 2010). Although use of these scales can and has provided relevant insights into the relation between character and reader, it remains questionable to what extent they reliably reflect the online and dynamic process of identification and to what extent this process is verbally reportable by its experiencer (see Jacobs, 2015b). Alternative measures of identification, both online and offline, could contribute to a more complete understanding of how viewpoint representations influence readers’ identification with narrative characters.

**Table 2** below shows how the various distinguished identification dimensions could be manipulated through variations in linguistic viewpoint representations as well as (neuro)psychological and psychophysiological methods that enable measurement of the effects of these representations on identification. The proposed measurements can be characterized in terms of the type of temporal relationship between story reading and measurement (online versus offline) and the type of inferential relationship between story experiences and measurement (indirect versus direct) as distinguished by Dixon and Bortolussi (2016). Specifically, methods that enable *online, indirect* assessments of identification (fMRI and measures of heart rate, galvanic skin response, and facial expressions) are proposed as a means to complement both indirect and direct offline measures and to gain more insight into the actual *process* of identification, whereas *offline, indirect* assessments of identification (virtual spatial navigation, picture recognition, implicit association, action-sentence compatibility) are proposed as a means to complement both online and offline direct self-report measures and to gain more insight into the actual *experience* of identification.

**Table 2:** Examples of the manipulation and measurement of the various identification dimensions.

Identification dimension	Linguistic manipulation	Measurement examples
(i) Spatiotemporal	(1a) Grammatical choices - Subject/object (1b) Tense - Past/present/plusquamperfectum (1c) Deictic elements - Proximal/distal	(1) Virtual spatial navigation assessment
(ii) Perceptual	(2a) Verbs of bodily sensation - Present/absent (2b) Verbs of perception - Present/absent	(2.1) fMRI (2.2) Picture recognition task
(iii) Cognitive	(3a) Verbs of cognition - Present/absent (3b) Metaphoric expressions of mental state - Present/absent (3c) Thought reports - Present/absent - Direct/indirect/free indirect	(3) fMRI
(iv) Moral	(4a) Evaluations - Present/absent - Positive/negative (4b) Attributions - Present/absent - Positive/negative	(4) Implicit association test
(v) Emotional	(5a) Verbs of emotion - Present/absent - Positive/negative (5b) Adjectives of emotion - Present/absent - Positive/negative (5c) Metaphoric expressions of emotional state - Present/absent - Positive/negative	(5.1) Galvanic skin response (5.2) fMRI (5.3) Heart rate variability (5.4) Facial expressions
(vi) Embodied	(6) Action verbs - Many/few - Concrete/abstract	(6.1) fMRI (6.2) Action-sentence compatibility paradigm

#### Measuring Spatiotemporal Identification

##### Virtual spatial navigation assessment

Virtual spatial navigation assessments provide an opportunity to measure readers’ spatiotemporal identification with narrative characters. In this type of assessment, participants are asked to find objects in a virtual 3D environment (Ventura et al., 2013). The time taken to fulfill this task is regarded as an indication of participants’ spatial ability. In the context of narrative identification, virtual spatial navigation tasks can be used to measure the extent to which readers adopt the spatiotemporal position of a character. For example, a narrative could describe various objects a character encounters while moving through an environment with few landmarks. After reading, participants could be shown a 3D setting of the narrative they can virtually move through and could be asked to find the various objects encountered by the character by copying the character’s route. This should take less time if the participant strongly identified with the character during reading, i.e., if the participant adopted the spatiotemporal position of the character.

In line with P1, we expect that the performance on a spatial navigation assessment is influenced by linguistic signals of a character’s spatiotemporal position. Specifically, we expect that people perform better after reading a story in which the character takes the grammatical subject (versus object) position. In addition, we expect that people perform better after reading a story described in the present tense or plusquamperfectum (versus the past tense). Finally, we expect that people perform better after reading a story featuring proximal (versus distal) deictic elements. Confirmation of these expectations would serve as an indication that linguistic signals of a character’s spatiotemporal position influence readers’ identification with that character.

#### Measuring Perceptual Identification

##### fMRI

A first method to measure perceptual identification involves the use of fMRI. Various studies have convincingly shown that if people imagine perceiving an object, brain areas are activated that overlap with those responsible for actual perception (see Sanford and Emmott, 2012). Imagining a character’s perceptions is likely to have similar effects and, in line with P2, this can be induced by linguistic signals of these perceptions. Specifically, we expect that readers show more activation in brain areas associated with sensory perception (e.g., visual cortex and auditory cortex) if narrative events are observed by the character as indicated by the presence (versus absence) of verbs of perception. Similarly, we expect that the presence (versus absence) of verbs of bodily sensation increases activation in the brain area associated with the specific sensation of the narrative’s character. For example, verbs expressing a prickling sensation are likely to increase activation of, among other areas, the anterior cingulate cortex and the secondary somatosensory cortex (e.g., Davis et al., 2002), whereas verbs expressing a painful bodily sensation are likely to increase activation of pain-sensitive regions such as the thalamus and the insula (e.g., Wager et al., 2004). Confirmation of these expectations would indicate that linguistic cues of a character’s perceptual viewpoint increase the reader’s perceptual identification with that character.

##### Picture recognition task

Perceptual identification can also be measured offline, by using picture recognition tasks. In this type of tasks, participants are asked to respond to names or pictures of objects by, for example, pressing buttons to indicate whether or not the objects were part of the narrative world. Yaxley and Zwaan (2007) employed a picture recognition task to assess readers’ representation of characters’ perceptual viewpoints. They manipulated the implied visibility of objects by having participants read sentences in which the character’s vision of an object was either clear (“Through the clean goggles, the skier could easily identify the moose”) or unclear (“Through the fogged goggles, the skier could hardly identify the moose”). Results showed that participants were faster to recognize clear pictures after reading sentences implying clear vision and faster to recognize unclear pictures after reading sentences implying unclear vision. These traces are not restricted to visual simulation but extend to auditory simulation as well in the sense that readers mentally simulate hearing the sounds they read about (Kaschak et al., 2006). The studies by Kaschak et al. (2006) and Yaxley and Zwaan (2007) indicate that descriptions of a character’s perceptions cause readers to mentally simulate those perceptions; they see and hear what the character sees and hears.

In studying the effects of perceptual viewpoint markers on identification, picture recognition tasks can be employed to assess the alignment between the perceptual viewpoints of character and reader. Although any description of an object is likely to induce a mental representation of that object (e.g., Zwaan et al., 2002), the mental representation can be expected to be strongest when the object is linguistically encoded as being observed by a character. Thus, in line with P2, we expect that readers are faster to recognize objects described in the narrative if those objects are perceived by the character as indicated by the presence (versus absence) of perceptual verbs. Again, confirmation of this expectation would indicate that perceptual viewpoint cues increase the reader’s identification with the character.

#### Measuring Cognitive Identification

##### fMRI

Effects of cognitive viewpoint cues on cognitive identification can be measured with fMRI techniques. A recent study measured activation of neural networks associated with mentalizing (i.e., networks responsible for understanding other persons’ beliefs, desires, and intentions) and networks associated with action and perception during narrative processing (Nijhof and Willems, 2015). Results showed, among other findings, that story parts describing characters’ mental activity led to an increased activation of mentalizing networks. Based on this finding, and in line with P3, we expect that linguistic expressions of a character’s viewpoint affect readers’ mentalizing processes. Specifically, we expect that readers show more activation in their medial prefrontal cortex when processing narratives representing a character’s mental state as signified by the presence (versus absence) of cognitive verbs, the presence (versus absence) of metaphoric expressions of a mental state, and the presence (versus absence) of thought reports. Confirmation of these expectations would indicate that linguistic cues of a character’s psychological viewpoint increase the reader’s cognitive identification with that character.

In addition, it can be expected that the mode of thought representation affects readers’ identification. In an fMRI study by Yao et al. (2011), participants silently read short stories including either direct or indirect speech reports. Results showed greater activation in voice-selective areas of the auditory cortex for direct (versus indirect) speech reports. This result indicates that readers mentally simulate hearing a character’s voice most strongly if its voice is rendered in a direct way (see also Yao and Scheepers, 2011). The mental simulation of a character’s thoughts can be measured in a similar way, by having participants read stories including characters’ thoughts that are reported in either direct or indirect mode. Activation of voice-selective areas of the auditory cortex would implicate that readers simulate the character’s *inner voice*. We expect that this simulation is strongest if this inner voice is linguistically encoded as a direct (versus indirect) thought. The free indirect mode, being grammatically and conceptually closer to the character (Sanders, 2010), is expected to render effects more similar to the direct representation mode than to the indirect mode.

#### Measuring Moral Identification

##### Implicit association tests

Implicit association tests (Greenwald et al., 1998) offer an interesting opportunity to measure readers’ moral identification with narrative characters. These tests are used to assess the (unconscious) associations people have with respect to all sorts of concepts and, for example, the degree to which they hold societal stereotypes. In an implicit association test, people are asked to sort attributes into two categories. Categorization is typically faster if the categories reflect associations held by the participant. For example, Greenwald et al. (1998) found that white American students were faster to sort attributes into a category in which White (Black) was paired with Pleasant (Unpleasant) compared to a category in which White (Black) was paired with Unpleasant (Pleasant). This result, replicated and extended in numerous studies, is taken as a reflection of unconsciously held associations, in this case racial stereotypes.

Implicit association tests can tap into associations that are difficult to detect by means of explicit self-report scales and, as such, have the potential to determine if and to what degree readers identify with narrative characters displaying behavior and attitudes that would generally be classified as undesirable or even immoral. Specifically, moral identification with a narrative character can be assessed by varying the categories such that these either match or mismatch associations held by the character. If moral identification takes place, categorization should be faster in a matching situation regardless of readers’ own prior associations. In line with P4, we expect that this effect is strongest after reading narratives with (versus without) explicit linguistic encoding of the character’s evaluations and attributions. Confirmation of this expectation would indicate that linguistic signals of a character’s moral viewpoint influence identification.

#### Measuring Emotional Identification

##### Galvanic skin response and heart rate variability

Readers’ emotional identification with narrative characters can be measured with psychophysiological methods. Galvanic skin response and heart rate variability are of specific interest since these measures indicate the extent to which a person is emotionally aroused. Both measures have been successfully employed in recent studies on narrative processing (e.g., Sukalla et al., 2015).

Measuring effects of emotional viewpoint cues on readers’ electrodermal activity and heart rate can reveal the extent to which these cues influence their identification with narrative characters. For example, in a study by Wallentin et al. (2011), participants listened to a story of which each line had been rated on emotional intensity while their heart rate was being measured. A positive correlation was found between emotionally intense parts of the story and participants’ heart rate variability, reflecting an increased emotional response during the processing of emotion-laden story parts. Based on this finding, and in line with P5, we expect that readers show stronger heart rate variabilities and, in addition, stronger skin responses when processing parts of narratives that represent a character’s emotional state as indicated by the presence (versus absence) of verbs of emotion, the presence (versus absence) of adjectives of emotion, and the presence (versus absence) of metaphoric expressions of a character’s emotional state. Confirmation of these expectations would indicate that linguistic cues of a character’s emotional viewpoint increase the reader’s emotional identification with that character.

##### fMRI

A second method to measure emotional identification involves the use of fMRI (see, e.g., Altmann et al., 2012; Hsu et al., 2015 for fMRI studies on the processing of emotion-laden narratives). In the study by Wallentin et al. (2011), the heart rate variability measure was combined with BOLD responses. Results showed that highly intense parts of the story were accompanied by activation in brain areas associated with emotional responses to auditory stimuli. Based on this finding, and in line with P5, we expect these regions to become activated while reading stories representing a character’s emotions as indicated by the presence (versus absence) of verbs of emotion, adjectives of emotion, and metaphoric expressions of a character’s mental state.

In addition, fMRI can be employed to measure effects of the manipulation of the valence of a character’s emotions. Jacobs et al. (2016a) propose the following neural networks to be relevant in measuring readers’ processing of emotion-laden story parts: the bilateral medial prefrontal cortex, the supramarginal gyrus/temporoparietal junction, the left dorsolateral prefrontal correct, and the left posterior middle temporal gyrus. It can be expected that the stronger readers identify emotionally with a character, the stronger the activation of these networks should be – dependent on the valence of the character’s emotions and the extent to which these emotions are linguistically encoded.

##### Facial expressions

Facial expression recognition software can be employed to measure readers’ emotions and to subsequently assess the level of correspondence with the character’s emotions, with high correspondence indicating a high level of identification. By measuring readers’ facial expressions, a crucial distinction can be made between more sympathetic or empathic responses (i.e., the reader feels *for* the character) and true identification (i.e., the reader feels *with* the character, as if *being* the character) (Coplan, 2004, 2006). For example, while reading a passage in which a character feels upset, readers’ faces might express that they feel sorry for the character or feel upset themselves. Whereas the former reaction would signal affective empathy, the latter would signal identification by means of the correspondence between the emotions of character and readers.

In line with P5, we expect correspondence between the reader’s and the character’s emotions to be highest when the character’s emotional viewpoint is linguistically encoded. Specifically, we expect that readers’ emotions show a higher level of correspondence to characters’ emotions in narratives that represent a character’s emotional state as indicated by the presence (versus absence) of verbs of emotion, the presence (versus absence) of adjectives of emotion, and the presence (versus absence) of metaphoric expressions of a character’s emotional state. In addition, correspondence between emotions could be assessed after manipulation of the valence of a character’s emotions. For example, narratives could be manipulated such that they represent either a character’s positive emotions or a character’s negative emotions, or –combining moral identification and emotional identification– such that readers experience positive emotions when a character fails in an amoral goal and experience negative emotions when a character succeeds in an amoral goal (see Raney, 2004). High levels of correspondence between the valence of the character’s emotions and the valence of the reader’s emotions would indicate high levels of identification.

#### Measuring Embodied Identification

##### fMRI

A first method to measure embodied identification is the use of fMRI. Various fMRI studies have established a link between reading action verbs and the mental simulation of actions (Hauk et al., 2004; Raposo et al., 2009). Since actions are central to narrative discourse, any narrative is likely to induce action simulation. However, in line with P6, action simulation can be expected to be strongest in stories featuring many (versus few) action verbs. In addition, the use of concrete (*she strolled into the garden*) versus abstract (*she went into the garden*) action verbs is likely to induce stronger action simulation. Confirmation of these hypotheses would lend support to the assumption that linguistic signals of a character’s actions influences readers’ embodied identification.

##### Action-sentence-compatibility tasks

A second method to measure readers’ embodied identification can be found in the use of action-sentence-compatibility tasks. In such tasks, participants read sentences that imply actions in a certain direction. For example, one study employed sentences implying a movement either away from the participant’s body (e.g., “Close the drawer”) or toward the participant’s body (e.g., “Open the drawer”) (Glenberg and Kaschak, 2002). Participants had to judge the semantic and grammatical correctness of the sentences by moving a button either away from or toward their body. Judgments were made faster in matching (versus mismatching) situations, i.e., if the movement implied by the sentence matched the movement the participants had to make.

Similar tasks can be used to measure readers’ embodied identification with narrative characters, for example by asking participants to read a story in which a character moves in a certain direction and to subsequently ask them to judge the accuracy of sentences describing narrative events after reading by pushing a button in a certain direction. According to P6, judgments should be made faster after reading a story with many (versus few) action verbs that match the required push direction. Confirmation of this expectation would indicate that the linguistic encoding of a character’s actions enhances embodied identification.

#### First versus Third Person Characters

We expect each of the hypothesized effects to occur in first person as well as third person stories. However, effects might be strongest in first person stories for two reasons. First, in constrast with third person stories, first person stories signal by definition an internal perspective (D’Ailly et al., 1995; Graesser et al., 2002). Second, various studies have shown that readers, given more options, identify most strongly with the character who is referred to by first person pronouns and whose inner consiousness is represented (De Graaf et al., 2012; Hoeken and Fikkers, 2014; Hoeken et al., 2016). Nevertheless, a recent study found that third person stories elicited slightly more arousal among readers, as indicated by a higher number of peaks observed in the electrodermal activity of their skin (Hartung et al., 2016). This seems to indicate that emotional identification is strongest in third person stories. In light of this finding, it is not implausible that the relative strength of the effects of viewpoint markers in first versus third person stories differs across the various dimensions of identification.

A series of experiments on perceived character transparency supports this line of reasoning (Kotovych et al., 2011). In this study, character transparency is conceptualized as one aspect of identification. It refers to the degree to which the thoughts and acts of a character are “clear and transparently understandable.” Results of the experiments showed that in first person stories, explicit information about the character’s beliefs and attitudes reduced character transparency, presumably because explicit information inhibits readers to draw their own inferences about the character, a process through which the perceived similarity with the character is likely to increase. By contrast, in third person stories, viewpoint markers representing the character’s thoughts in free indirect mode were found to enhance character transparancy. Based on these results, it might be expected that the effects of viewpoint markers on the various dimensions of identification is different for first versus third person stories.

Importantly, in third person narratives, the extradiegetic narrator has more opportunities to create gaps between what readers know about the main character and what the main character itself knows. Needless to say, such gaps can create great anticipation and tension effects in the reader, both in positive (promise of gain and joy) and in negative (threat of danger and loss) sense. Such identification effects could also very well be measured by bodily reactions as well as fMRI. However, a detailed account of these effects falls beyond the scope of this study.

## Conclusion and Discussion

The present paper started out by addressing two issues in current research on identification with narrative characters: (1) the use of offline, self-report scales to measure afterward the dynamic online process of identification and (2) the lack of knowledge about the linguistic elements that guide identification, resulting from a lack of studies systematically manipulating these elements. We presented a multidimensional Linguistic Cues Framework that might be beneficial in overcoming these issues and developed a research agenda incorporating linguistic and narratological accounts of viewpoint in discourse and psychological and neurocognitive methods suitable for measuring effects of these viewpoint elements on identification. To further advance future studies in this direction, some final considerations are in place.

First, the framework connects various types of linguistic viewpoint indicators to various dimensions of identification. These connections were in our overview distinguished for the sake of the argumentation; it is important to note, however, that a clear-cut one-to-one relation between the distinctive viewpoint cues and the various identification dimensions is unlikely to be found in real narratives. They will be found together, in various combinations and degrees, for the reason that in natural narrative discourse, the linguistic expressions evoking identification cannot be used in isolation. These expressions appear in combination and closely cooperate to establish a stronger or lesser degree of identification of a particular kind: experiential, emotional, attitudinal, or all of the above. For example, the use of perceptual viewpoint cues might evoke not only perceptual identification but also a certain degree of emotional identification, in particular when the reported perceptions trigger personal memories. Similarly, the use of cognitive viewpoint cues might evoke not only cognitive identification, but also a certain degree of perceptual identification, for example when a thought report includes references to a character’s senses. Nevertheless, it is reasonable to assume that the main burden for evoking emotional identification is carried by verbs and adjectives of emotion; that cognitive identification is mainly – but not exclusively – affected by thought reports and cognitive verbs; that perceptual identification is mainly – but not exclusively – affected by verbs of perception and bodily sensation; et cetera. This assumption could be empirically tested by employing and combining the methods to measure the various distinctive identification dimensions as outlined in this paper.

Second, it is essential to distinguish between, on the one hand, perspective-taking as an (unconscious) act during the representation of narrative discourse, resulting from linguistic text characteristics in the six dimensions which guide readers toward the representation of subjective viewpoints on events and situations; and, on the other hand, identification with a particular subject in the narrative discourse as a process, consequential of multiple, pervasive perspective-taking of this subject. Thus, while the relevant linguistic cues identified in this paper steer, in essence, perspective-taking processes, we argue that identification is to be understood as a result of these processes. In a narrative with several characters, repetitive perspective-taking with a particular character, guided by linguistic characteristics of various perspective dimensions (e.g., spatial, emotional, and cognitive perspective), will induce readers to identify more strongly with this character rather than with other optional characters. Note that this is not to say that identification is influenced by linguistic characteristics of perspective alone. Identification can also be guided by factors such as the degree to which readers experience that the demographics, personality treats, attitudes, behavior, and/or socio-cultural context of a particular character are like them, or are likeable (e.g., Hoeken and Sinkeldam, 2014); the cultural background of readers and the degree to which this induces them to identify with narrative characters in general (e.g., Leung and Cohen, 2011); and the instruction or intention which guides readers in their reading and interpretation process (e.g., Sestir and Green, 2010). All of these factors relevant to identification can be explained by an evolutionary developed human preference for narrative as a means to represent other people’s experiences, to persuade others of particular views and convictions, and to simulate and even ‘train’ unexpected and dangerous situations, in order to experience what consequences of particular choices would be without being at real risk (Boyd, 2009).

Third, studies testing the impact of linguistic characteristics of perspective as outlined in this paper would have to ensure clean manipulations of viewpoint cues while controlling for other text-linguistic features that can affect readers’ behavioral, psychophysiological, and neuronal responses to stories (e.g., Jacobs, 2015a; Jacobs et al., 2016b). For example, Lehne et al. (2015) found that reading suspenseful passages resulted in activation of brain areas associated with cognitive theory-of-mind processes. A factor like suspense should therefore be taken into account when studying effects of cognitive viewpoint cues on cognitive identification, which are in the present paper hypothesized to be observable in theory-of-mind networks. Previous studies have succeeded in manipulating viewpoint cues while controlling for confounding factors (Van Krieken and Sanders, 2017) and, importantly, in manipulating these cues in natural stories (Sanders and Redeker, 1993). Thus, studies assessing the impact of viewpoint cues on identification can adhere to standards of internal as well as external validity, the latter of which being one of the central aims within the ‘new’ scientific study of literature (Burke, 2015; Willems and Jacobs, 2016).

Such studies could also mark a step forward in tackling the difficulty of inferring the occurrence of mental processes taking place while reading stories from observed brain activation. In a recent debate on methodological issues involved in the scientific study of literary experiences, both Jacobs (2015b) and Kuiken (2016) underscore how this so-called ‘reverse inference’ problem threatens the validity of neurocognitive studies on reading experiences. As Jacobs (2015b) argues, careful text manipulations are a prerequisite to avoid the reverse inference fallacy. The hypotheses put forward in the present paper predict specific effects on the online measures of specific linguistic variations. As such, any differences in these measures are easier to interpret compared to a case in which variations in online measures need to be traced back to variations in the stimulus materials; moreover, results on online measures of identification are in this approach easier to relate to offline measures of identification.

With proper consideration of the issues discussed above, research testing the framework presented in this paper would advance our understanding of identification processes in several important respects. First, studies employing fMRI techniques and psychophysiological measures would provide insight into the strength and duration of identification processes as they evolve *during* narrative processing, thus complementing insights produced by studies employing offline measures (e.g., Moyer-Gusé and Nabi, 2010; Tal-Or and Cohen, 2010). Second, research in this direction would enable a comparison between explicit self-report measures and more implicit measures (e.g., picture recognition tasks and implicit association tests), thus shedding light on the question as to what degree processes of narrative engagement are verbally reportable by readers (Jacobs, 2015b). Third, it would offer an empirical test of the assumption that identification is a multidimensional experience (Cohen, 2001, 2006) and reveal the extent to which these dimensions are independent or, conversely, the extent to which these dimensions are evoked as interactions. Fourth, it would provide valuable additions to the existing literature by scrutinizing which linguistic characteristics of narratives prompt and guide identification processes. Although these processes are unlikely to be influenced solely by viewpoint markers, studying their effects in isolation would make a valuable contribution to our understanding of the relation between the linguistic characteristics of stories and the degree to which readers identify with characters.

In broader terms, research in this direction has the potential to provide answers to some of the most fundamental questions about human communication. Although stories have long been recognized as the dominant mode in which people exchange information (e.g., Barthes, 1977; Gottschall, 2012), the question remains what it is exactly that makes their use so attractive and their impact so powerful (e.g., Green, 2008; see also Dixon and Bortolussi, 2016). Examining connections between the linguistic form of narratives and readers’ physical, psychological, and neurocognitive responses to narratives will contribute to a clearer understanding of how and why people can become so deeply involved with characters whose lives merely exist by the virtue of written words.

## Author Contributions

All authors listed have made a substantial, direct and intellectual contribution to the work, and approved it for publication.

## Conflict of Interest Statement

The authors declare that the research was conducted in the absence of any commercial or financial relationships that could be construed as a potential conflict of interest. The handling Editor declared a shared affiliation, though no other collaboration, with one of the authors HH and states that the process nevertheless met the standards of a fair and objective review.
